# Chrysophanol Inhibits NALP3 Inflammasome Activation and Ameliorates Cerebral Ischemia/Reperfusion in Mice

**DOI:** 10.1155/2014/370530

**Published:** 2014-04-29

**Authors:** Nan Zhang, Xiangjian Zhang, Xiaoxia Liu, Hong Wang, Jing Xue, Jingying Yu, Ning Kang, Xiaolu Wang

**Affiliations:** ^1^Department of Neurology, Second Hospital of Hebei Medical University, Shijiazhuang, Hebei 050000, China; ^2^Hebei Collaborative Innovation Center for Cardio-Cerebrovascular Disease, Shijiazhuang, Hebei 050000, China; ^3^Hebei Key Laboratory for Neurology, Shijiazhuang, Hebei 050000, China

## Abstract

The most effective way to contain cerebral ischemic injury is reperfusion; however, reperfusion itself may result in tissue injury, for which inflammatory damage is one of the main causative factors. NALP3 inflammasome is a multiprotein complex. It consists of NALP3, ASC, and caspase-1, whose function is to switch on the inflammatory process. Chrysophanol is an extract from plants of Rheum genus and it possesses many pharmacological effects including its anti-inflammation activity. In this study, the effects of chrysophanol in cerebral ischemia/reperfusion and the potential mechanisms were investigated. Male CD1 mice were subject to transient middle cerebral artery occlusion (tMCAO). The NALP3 inflammasome activation status and its dynamic expression during the natural inflammatory response induced by tMCAO were first profiled. The neuroprotective effects of chrysophanol were then assessed and the potential mechanisms mediating the observed neuroprotection were then explored. Physical parameters including neurological deficit, infarct size, brain edema, and BBB permeability were measured at 24 h after tMCAO. Confocal microscopy, Western blotting, immunohistochemistry, and qRT-PCR techniques were utilized to analyze the expression of NALP3 inflammasome and IL-1**β**. Our results indicated that the brain tissue damage during cerebral ischemia/reperfusion is accompanied by NALP3 inflammasome activation. Chrysophanol could inhibit the activation of NALP3 inflammasome and protect cerebral ischemic stroke.

## 1. Introduction

Stroke constitutes one of the major causes leading to disability and death worldwide [1]. Inflammatory response has been confirmed to play a detrimental role in the pathogenesis of cerebral ischemia/reperfusion (I/R) injury [2]. The relationship between inflammation and cerebral I/R outcome has assured the considerable and continued interest in the development of anti-inflammation oriented therapies to mitigate I/R-induced brain damage.

The inflammatory process involves activation and interaction of a broad spectrum of immune factors pioneered by the upregulation of proinflammatory cytokines. These cytokines, including members of the interleukin (IL)-1 family such as IL-1*β*, are detected in the infarct area and their imbalance is associated with brain tissue damage. A previous study has demonstrated that mice deficient in IL-1*β* show improved survival, reduced the infarct volume, and improved neurological functions as compared with wild-type mice after middle cerebral artery occlusion (MCAO) [3].

**Figure 1:**
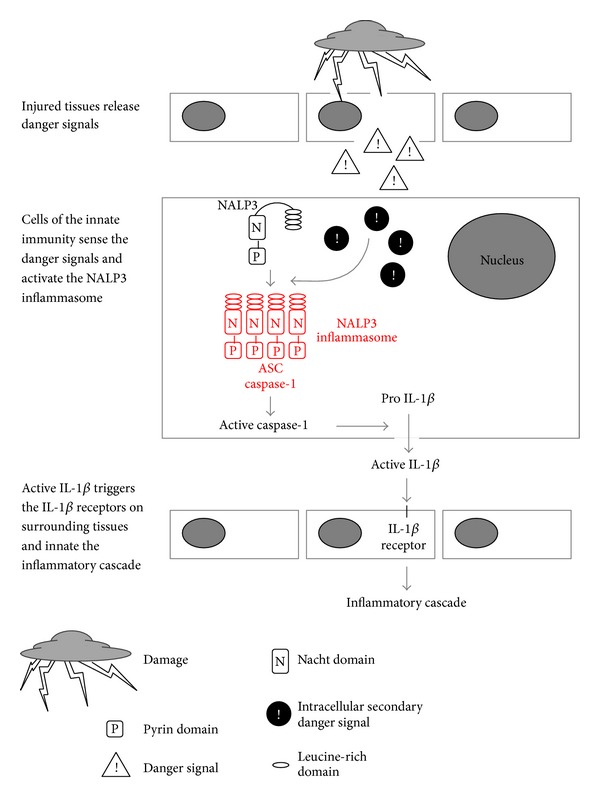
Model of danger signals activation of the NALP3 inflammasome. Tissue injury leads to the formation and release of danger signals such as ATP or uric acid crystals that are recognized by the innate immune system. A number of these signals mediate a potassium efflux or other secondary intracellular danger signals that are required for NALP3 inflammasome activation [51, 52]. NALP3 inflammasome then oligomerizes to recruit the adaptor ASC and caspase-1 [53]. Activation of caspase-1 results in the processing and maturation of pro IL-1*β* into its biologically active form, active IL-1*β* [12, 54]. Active IL-1*β* will then trigger the IL-1*β* receptor, leading to the activation of multiple cytokines involved in the inflammation cascade [55].

Recent evidence suggested that the downstream processing of IL-1*β* is regulated by some cytosolic factors such as inflammasomes, a family of protein complexes that were recently identified as the cellular machinery responsible for recognizing pathogen-associated molecular patterns and reacting to these through activation of inflammatory processes [4]. Among different types of inflammasomes is the NALP3 inflammasome, which has been well characterized in a variety of mammalian cells. It is characterized as a proteolytic complex mainly composed of the NACHT domain-, leucine-rich repeat-, and pyrin domain (PYD)-containing protein 3 (NALP3), the adaptor protein apoptosis-associated speck-like protein (ASC), and caspase-1 [5]. The NALP3 inflammasome can be activated by bacterial toxins [6] or pathogen-associated molecular patterns, such as muramyldipeptide, and other stimuli. NALP3 can also detect and respond to endogenous stress-associated danger signals, such as ATP [7], ROS [8], monosodium urate crystals [9], low intracellular potassium concentrations, sodium overload [10], or *β*-amyloid by activating caspase-1, and active caspase-1 subsequently matures the proinflammatory IL-1*β* family cytokines by cleaving their proforms into biologically active cytokines in turn [11, 12]. Active IL-1*β* then triggers the IL-1*β* receptors on surrounding tissues [13, 14], leading to the activation of multiple cytokines involved in the inflammation cascade, including IL-8, TNF, and IL-17 [15, 16] (Figure 1). However, the role of NALP3 inflammasome in cerebral I/R inflammatory disorders has not been well explored [17, 18].

**Figure 2:**
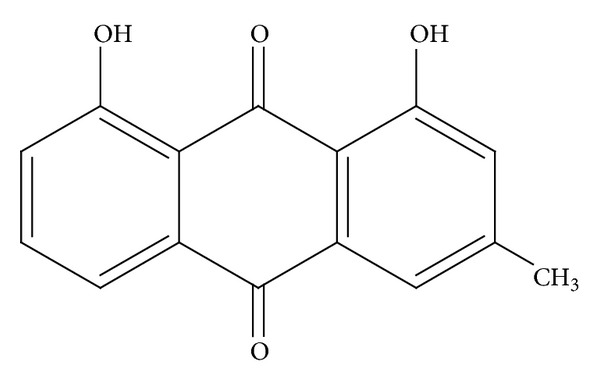
The chemical structure of CHR.

Chrysophanol (CHR), a member of the anthraquinone family, was originally extracted from plants of Rheum genus (Figure 2). CHR has been shown to have multiple pharmacological effects, including its anti-inflammation activity, for which the underlying mechanisms remain to be elucidated. Previous studies have shown that CHR inhibits caspase-1 and its downstream cytokines expression in colitis [19]. However, little is known on the relationship between CHR and NALP3 inflammasome during cerebral I/R.

Using mouse transient middle cerebral artery occlusion (tMCAO) model, this study was designed to examine the cellular distribution and dynamic expression of NALP3 inflammasome during cerebral I/R and investigate whether CHR has any neuroprotective effect and what are the underlying mechanisms mediating this protection if it does have.

## 2. Methods

### 2.1. Experimental Animals

Male CD1 mice (25~30 g) were purchased from the Vital River Laboratory Animal Technology Co., Ltd., Beijing, China. All mice were given at least 2 days to acclimatize ahead of any experimentation. During this period, all mice have free access to food and water. Animal houses were maintained in a 12/12 h light/dark cycle with humidity of 60% ± 5% and ambient temperature of 22°C ± 3°C. The experimental procedures were approved by the local experimental ethics committee as well as the institutional animal care and use committee.

### 2.2. Animal Surgery for tMCAO

The mouse tMACO model was established by following the surgical procedures as previously described [20, 21]. In brief, animals were anesthetized with chloral hydrate (350 mg/kg, intraperitoneally). Loss of righting reflex was affirmed before performing midline skin incision. The right common carotid artery (CCA), external carotid artery (ECA), and internal carotid artery (ICA) were then exposed through blunt separation. Branches of ECA were then cauterized and the ECA was ligated and cut off at the distance of 2 mm from bifurcation of CCA, while middle cerebral artery (MCA) was occluded by inserting a heparin-dampened monofilament nylon suture (Beijing Sunbio Biotech Co., Ltd., Beijing, China) into the ICA, which was moved forward until the flow of right MCA was reduced to 20%~30% of basal flow, which was monitored by a blood flow monitor (moor VMS-LDF, Moor Instruments Ltd., UK). One hour later, the filament was gently withdrawn for the reperfusion. Sham-operated mice underwent the same procedures except inserting a filament. The animals' body temperature was also monitored and maintained at 36.5°C to 37.5°C during experiment [22–24].

### 2.3. Experimental Groups and Treatments

All animals were divided into two groups: the control group and the drug treatment group. For the control group, a total of 63 mice were randomly divided into 7 subgroups, 9 mice each, including the Sham controls and untreated controls for six time points after tMCAO (3 h, 6 h, 12 h, 24 h, 48 h, and 72 h). For the treatment group, 162 mice were randomly divided into 6 groups: (1) the Sham group: animals received Sham operation and equal volume of 0.9% NaCl; (2) tMCAO group: animals received tMCAO and equal volume of 0.9% NaCl; (3) Vehicle group: animals underwent tMCAO and equal volume of 1% DMSO and 1% Tween-80 prepared with 0.9% NaCl; and (4) three CHR groups: animals underwent tMCAO and were treated with a high dose of CHR, (10 mg/kg, CHR-H) or middle dose of CHR (1 mg/kg, CHR-M) and low dose of CHR (0.1 mg/kg, CHR-L). Mice were reanesthetized and sacrificed at 24 h after tMCAO. The CHR product (Nanjing Zelang Medical Technological Co., Ltd., Jiangsu, China) with purity of more than 98% was first dissolved in 0.9% NaCl including 1% DMSO and 1% Tween-80 prior to the treatments. Drug or solvent was injected intraperitoneally 30 minutes prior to tMCAO operation.

### 2.4. Neurological Deficit Score

The infarct volume, brain edema, and blood brain barrier (BBB) permeability were determined by an examiner blinded to the experimental grouping at 24 h post-tMCAO for the mice from the drug treatment group. Meanwhile, a neurological test was administered on these animals following a scoring system adapted from the one developed by Longa et al. [20] as follows: 0, no deficits; 1, difficulty in fully extending the contralateral forelimb; 2, unable to extend the contralateral forelimb; 3, mild circling to the contralateral side; 4, severe circling; and 5, falling to the contralateral side.

### 2.5. Brain Infarct Volume

Infarct volume of brain was measured at 24 h after tMCAO. After the brains (*n* = 6 in each group) were dissected, each brain sample was cut into five slices with 2 mm thickness, incubated by a 2% solution of 2, 3, 5-triphenyltetrazolium chloride (TTC) at 37°C for 15 min, followed by fixation with 4% paraformaldehyde. With TTC staining, all normal tissue was stained in dark red, while the infarct area was stained in a pale gray color. All TTC-stained slices were then photographed and analyzed using Image-Pro Plus 5.1 software (Media Cybernetics, Inc., Bethesda, USA), and the infarct volumes were calculated as follows [25]: percentage hemisphere lesion volume (%HLV) = {[total infarct volume − (volume of intact ipsilateral hemisphere − volume of intact contralateral hemisphere)]/contralateral hemisphere volume} × 100%.

### 2.6. Brain Water Content

Brain water content was determined using the standard wet-dry method [26]. Mice (*n* = 6 in each group) were decapitated under deep anesthesia at 24 h post-tMCAO, while the cerebrum was removed and placed on a preprepared dry tray as quickly as possible. The frontal pole with 2 mm thickness and cerebellum were removed; only coronal slices with approximately 4 mm in thickness were left. The slices were divided into ischemic and nonischemic hemispheres. At the same time, the two hemisphere slices were packaged, respectively, with tin foil, and their wet weights were evaluated with electronic balance and then dried for 24 h at 100°C to get dry weights. Brain water content (BW) was calculated as follows: BW = (wet weight − dry weight)/wet weight × 100%.

### 2.7. Evans Blue Extravasation

At 2 h before the mice were anesthetized, 4 mL/kg of 2% Evans blue (Sigma) prepared with 0.9% NaCl was administered by tail vein injection. Then, they were perfused into heart with 0.9% NaCl. For quantitative measurement of Evans blue extravasation, the ischemic and nonischemic hemispheres were removed and homogenized in 1 mL of trichloroacetic acid and then centrifuged at 21,000 g for 20 minutes. Evans blue concentration was quantitatively determined by measuring the absorbance of the supernatant at 610 nm using spectrometer (infinite m200 pro, TECAN, Austria) [27].

### 2.8. Confocal Microscopy

Representative coronal brain sections of mice were obtained at 24 h after Sham operation or tMCAO (*n* = 3 for each group). Mice were perfused with 0.9% NaCl quickly followed by 4% paraformaldehyde in PBS. Fixed frozen cerebral sections (30 *μ*m thick) were blocked with 10% goat serum for 1 h and then incubated overnight at 4°C with primary antibodies from Abcam including anti-NALP3 antibody (1 : 100); anti-ASC antibody (1 : 100); anti-caspase-1 antibody (1 : 100); and anti-IL-1*β* antibody (1 : 100), together with anti-NeuN (1 : 200, Sternberger Monoclonal Incorporation, Lutherville, MD, USA). After 3 washes with PBS, they were incubated with secondary antibodies (1 : 100, Zhongshan Biology Technology Company, China) at 37°C for 2 h. Immunofluorescence was visualized using a Laser Scanning Confocal Microscope (Olympus FV10-ASW, Japan).

### 2.9. Western Blotting

All proteins were obtained from the cortex of the ischemic hemisphere using a total protein extraction kit from Applygen Technologies Inc., (Beijing, China) following the manufacturer's protocols. The protein concentrations of the extracts were determined using a BCA Protein Assay reagent kit (Novagen, Madison, WI, USA), while 50 *μ*g of proteins (*n* = 3 per group per time point) was separated by SDS/PAGE electrophoresis and transferred to PVDF membranes. The membranes were blocked with 5% nonfat milk in 0.01 M PBS at room temperature for 1 h and then incubated overnight at 4°C with the primary antibodies obtained from Abcam, including anti-NALP3 antibody (1 : 1000), anti-ASC antibody (1 : 1000), anti-caspase-1 antibody (1 : 500), and anti-IL-1*β* antibody (1 : 1000). Polyclonal rabbit anti-beta actin antibody (1 : 500) from Zhongshan Biotechnology was used for internal control. After three washes with TPBS, all membranes were incubated with IRDye 800-conjugated goat anti-rabbit or anti-mouse secondary antibodies for 1 h at room temperature. An imaging densitometer (LI-COR Bioscience) was used to analyze the relative density of each band.

### 2.10. Immunohistochemistry

For immunohistochemical staining, brain tissues were removed and immediately immersed in 4% paraformaldehyde with PBS for 24 h at 4°C. Brain sections (5 *μ*m thick) were blocked in 3% H_2_O_2_, 3% normal goat serum, and then incubated overnight with anti-mouse NALP3, ASC and IL-1*β* rabbit polyclonal antibodies (1 : 100, Abcam Biotechnology), and anti-mouse caspase-1 rabbit polyclonal antibody (1 : 100, Sigma Biotechnology). The secondary antibodies, secondary biotinylated conjugates, and diaminobenzidine were from the Vect ABC kit (Zhongshan Biology Technology Company, China). In the end, 20 cross sections were taken from each mouse brain for immunostaining. The cause is four factors will be observed altogether with each bearing 5 sections. Meanwhile, five visual fields of ischemic region from the infarct area were selected and the immunoreactive cells were counted under a 400x light microscope (*n* = 3 per group per time point).

### 2.11. Quantitative Real-Time Polymerase Chain Reaction (qRT-PCR)

Total RNA from ischemic region cortex was isolated using Trizol reagent (Invitrogen, Carlsbad, CA, USA) and was reverse-transcribed into cDNA using Revert Aid first Strand cDNA Synthesis kit (Fermentas International Inc., Burlington, Canada) for Quantitative PCR (MX 3005P, USA) in the presence of a fluorescent dye (SYBR Green I; Cwbio). Relative abundance of mRNA from the perspective target genes was calculated after normalization to *β*-actin RNA and was calculated by the 2^−ΔΔCt^ method. Real-time PCR was used to analyze the levels of NALP3, ASC, caspase-1, and IL-1*β* mRNA at 24 h after tMCAO. All primer sequences are provided in Table 1.

### 2.12. Statistical Analysis

All data were analyzed using proper tools from SPSS 13.0 package, while all quantitative data were expressed as mean ± SD. Statistical comparisons were conducted using one-way ANOVA followed by SNK and LSD tests for intergroup comparisons. For neurological deficit, Mann-Whitney *U* test was applied for comparisons between groups.

## 3. Results

### 3.1. NALP3, ASC, Caspase-1, and IL-1*β* Were in Neurons

**Figure 3:**
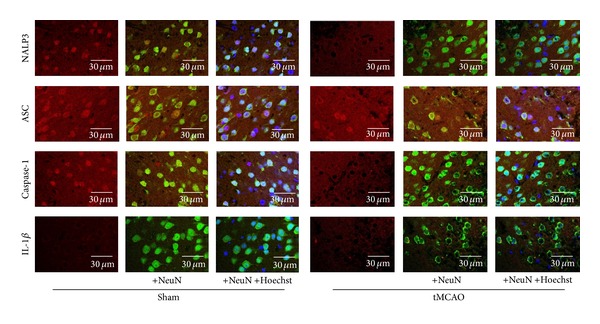
Cellular distribution of NALP3 inflammasome components and IL-1*β* in the ischemic penumbra zone of cerebral cortex. Sections were triple-stained for NALP3, ASC, caspase-1 and IL-1*β* (red), NeuN (green), and nucleus (blue). In tMCAO group, the neuronal karyopyknosis and chromatin margination could be observed and there was a small amount of neutrophils in marginal zone of infarction. In the normal brain (Sham), caspase-1 and NALP3 were mainly present in the nucleus of neurons whereas IL-1*β* was mainly localized in the cytoplasm. ASC was present in both the nucleus and cytoplasm. After tMCAO, NALP3 and caspase-1 were redistributed predominantly to the cytoplasm, while ASC and IL-1*β* location remained unchanged.

Confocal microscopy was utilized to detect the presence and the cellular distribution of NALP3 inflammasome components as well as IL-1*β* in the ischemic penumbra zone of the cerebral cortex. The neuronal karyopyknosis and chromatin margination could be observed and there was a small amount of neutrophils in the marginal zone of infarction. In the normal brain (Sham), caspase-1 and NALP3 were mainly localized in the nucleus of neurons, whereas IL-1*β* was mainly present in the cytoplasm. ASC was present in both the nucleus and cytoplasm. However, after tMCAO, NALP3 and caspase-1 were redistributed predominantly to the cytoplasm, while ASC and IL-1*β* locations remained unchanged (Figure 3).

#### 3.1.1. NALP3, ASC, Caspase-1, and IL-1*β* Were Upregulated during Cerebral I/R

**Figure 4:**
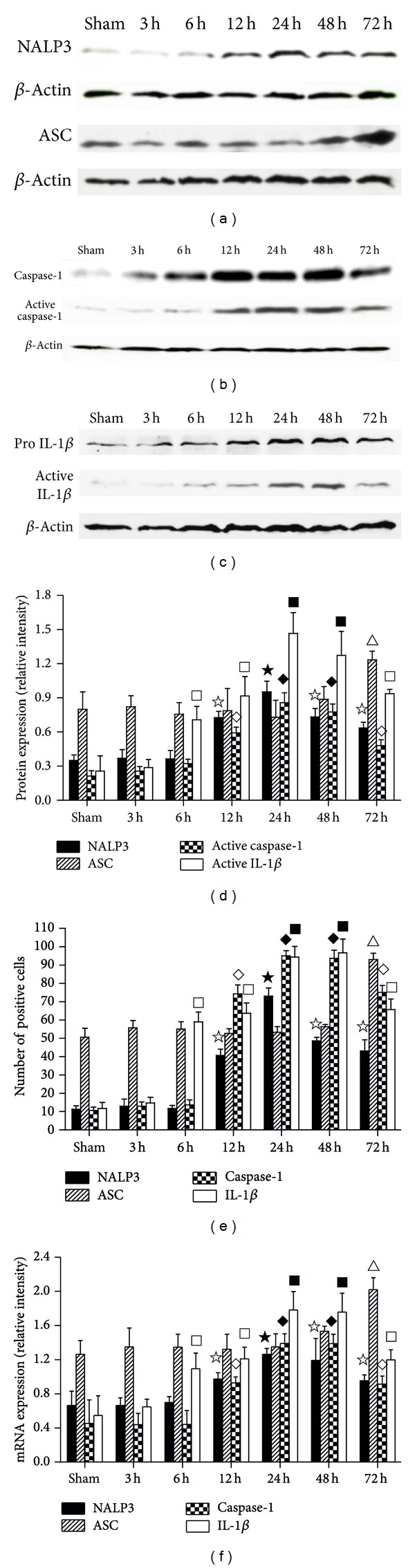
The dynamic expression of NALP3 inflammasome and IL-1*β* during the natural inflammatory response induced by tMCAO. Western blot of dynamic expression ((a), (b), (c)). Bar graph illustrating the dynamic expression of NALP3, ASC, active caspase-1, and active IL-1*β* in brain tissues (d). Bar graph illustrating dynamic expression of NALP3, ASC, caspase-1, and IL-1*β* as determined by immunohistochemistry (e). Bar graph illustrating the mRNA dynamic expression of NALP3, ASC, caspase-1, and IL-1*β* (f). For NALP3, ^*☆*^ *P* < 0.05 versus Sham, ^★^ *P* < 0.05 versus Sham, 3 h, 6 h, 12 h, 48 h, and 72 h. For ASC, ^△^ *P* < 0.05 versus Sham. For active caspase-1 and active IL-1*β*, ^◊^ *P* < 0.05 and ^□^ *P* < 0.05 versus Sham, ^*◆*^ *P* < 0.05 and ^■^ *P* < 0.05 versus Sham, 3 h, 6 h, 12 h, and 72 h.

Immunohistochemistry, qRT-PCR, and Western blotting were used to examine expression of NALP3, ASC, caspase-1, and IL-1*β* in brain tissue after tMCAO. The results showed that NALP3 and active caspase-1 were both upregulated as compared with Sham group (*P* < 0.05), beginning at 12 h and peaking at 24 h (Figures 4(a), 4(b), 4(d), 4(e), and 4(f)). In contrast, active IL-1*β* was also shown to be upregulated but with an earlier onset at 6 h post-tMCAO. ASC remained unchanged until 72 h after tMCAO (Figures 4(c), 4(d), 4(e), and 4(f)). Therefore, we took the time point of 24 h after tMCAO to observe the effects of CHR, so as to demonstrate the protective effects of CHR on acute cerebral I/R.

#### 3.1.2. CHR Reduced Neurological Deficits

**Figure 5:**
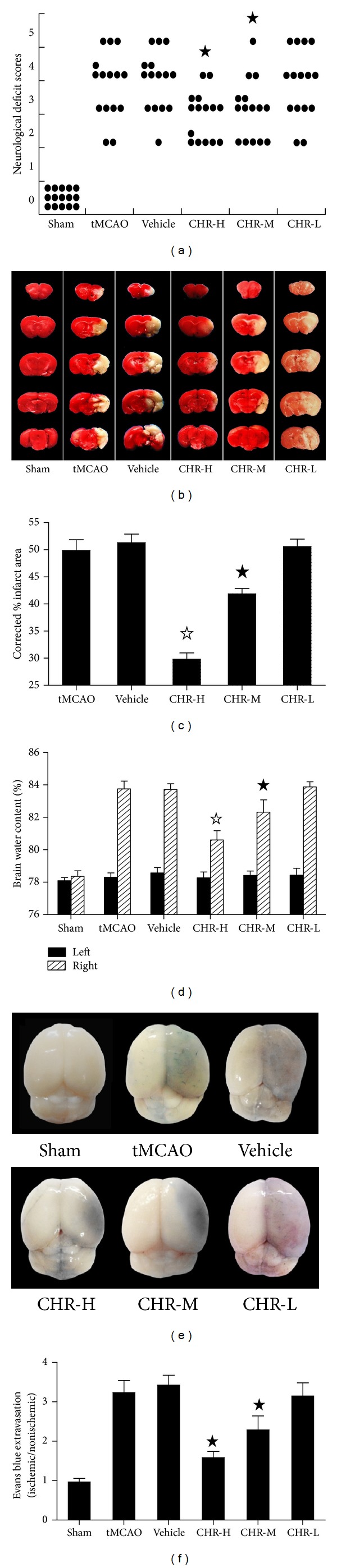
Effect of CHR on neurological deficit. The behavioral scores were significant reduced (^★^ *P* < 0.05 versus tMCAO and Vehicle, Mann-Whitney U test) (a). The infarct volume was reduced ^(*☆*^ *P* < 0.05 versus tMCAO, Vehicle and CHR-M, ^★^ *P* < 0.05 versus tMCAO and Vehicle) (b, c). The water content of ischemic hemisphere was reduced (^*☆*^ *P* < 0.05 versus tMCAO, Vehicle and CHR-M, ^★^ *P* < 0.05 versus tMCAO and Vehicle) (d). BBB permeability was ameliorated ^(★^ *P* < 0.05 versus tMCAO and Vehicle) (e, f).

Neurological deficit was examined and scored on a 6-point scale at 24 h after tMCAO and Mann-Whitney *U* test analysis was conducted. As depicted in Figure 5(a), mice in Sham group showed a neurological score as zero while the mice from the tMCAO and Vehicle groups showed expected higher neurological deficit scores after the surgery as compared with Sham group (*P* < 0.05). No significant difference was observed between the animals from tMCAO and Vehicle groups (*P* > 0.05). Remarkably, mice from the CHR-H and CHR-M groups showed significantly improved neurological function scores as compared with tMCAO and Vehicle groups after tMCAO (*P* < 0.05). By contrast, there was no significant effect in CHR-L group compared with tMCAO and Vehicle groups (*P* > 0.05).

#### 3.1.3. CHR Reduced the Infarct Volume

Infarct volume of each group was measured by TTC at 24 h after tMCAO (Figure 5(b)). Normal tissue was stained in dark red, while the infarct area appeared pale gray. No infarction was observed in Sham group. An extensive lesion was found in both cortex and striatum in animals from tMCAO and Vehicle groups (*P* < 0.05 versus Sham group). Nonetheless, there were no significant differences between tMCAO and Vehicle groups (49.87% ± 1.99% versus 51.31% ± 1.57%, *P* > 0.05). The neuroprotective effects of CHR at different doses were clearly observed by both TTC staining (Figure 5(b)) and examining the infarct volume at 24 h (Figure 5(c)). A significant protective effect from CHR was observed as the infarct volumes from animals in the CHR-H and CHR-M groups but not the CHR-L group are significantly different from the tMCAO and Vehicle groups (Figure 5(c)). There was a significant reduction in infarct volume in CHR-H (29.80% ± 1.15% versus 49.87% ± 1.99% and 51.31% ± 1.57%, *P* < 0.05) and CHR-M (41.86% ± 0.98% versus 49.87% ± 1.99% and 51.31% ± 1.57%, *P* < 0.05) groups as compared with tMCAO and Vehicle groups.

#### 3.1.4. CHR Reduced the Brain Edema

Wet-dry method was used as index to measure brain edema [26]. The measurements of the brain water content of the normal and the ischemic hemispheres of all experimental groups were shown in Figure 5(d). It was demonstrated that there was no change in brain edema in the Sham group but rather a significant increase in brain water content in the ischemic hemispheres in the tMCAO and Vehicle groups as compared with those from the Sham group (*P* < 0.05), while no statistic differences were observed between the tMCAO and Vehicle groups (83.75% ± 0.48% versus 83.72% ± 0.35% *P* > 0.05). Following CHR treatment, as compared with tMCAO and Vehicle groups, there were significant reductions in brain water content in the CHR-H group (80.60% ± 0.58% versus 83.75% ± 0.48% and 83.72% ± 0.35%, *P* < 0.05) and the CHR-M group (82.15% ± 0.68% versus 83.75% ± 0.48% and 83.72% ± 0.35%, *P* < 0.05). Again, no significant difference was observed between the CHR-L group and the tMCAO and Vehicle groups (83.88% ± 0.31% versus 83.75% ± 0.48% and 83.72% ± 0.35%, *P* > 0.05).

#### 3.1.5. The Amelioration of BBB Permeability

To evaluate BBB permeability at 24 h after tMCAO, BBB leakage was measured using Evans blue extravasation (Figures 5(e) and 5(f)). The blue area shows the extravagated Evans blue, indicating BBB disruption. As expected, an extensive BBB disruption was found in animals from the tMCAO and Vehicle groups (*P* < 0.05 versus Sham group), whereas no significant difference was observed between the tMCAO and Vehicle groups (*P* > 0.05) (Figure 5(f)). The protective effects of CHR with different doses were also observed by examination of Evans blue content at 24 h and, as compared with the tMCAO and Vehicle groups, significantly lower Evans blue extravasation was observed in the CHR-H and CHR-M groups (*P* < 0.05 versus tMCAO and Vehicle) (Figure 5(f)). In agreement with neurological deficit, infarct volume, and brain edema measurement, the CHR-L group did not show a significant difference in comparison with the tMCAO and Vehicle groups (*P* > 0.05).

#### 3.1.6. CHR Suppressed the Expression of NALP3, Caspase-1, and IL-1*β*

**Figure 6:**
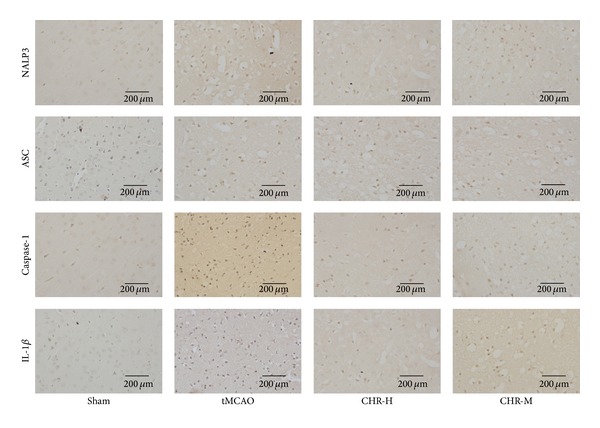
Immunohistochemical staining of NALP3, ASC, caspase-1, and IL-1*β* in the cerebral cortex at 24 h after ischemia (400x magnification). As compared with tMACO and Vehicle groups, the expression of NALP3, caspase-1, and IL-1*β* was significantly reduced in CHR-H and CHR-M groups, but the expression of ASC remained unchanged.

**Figure 7:**
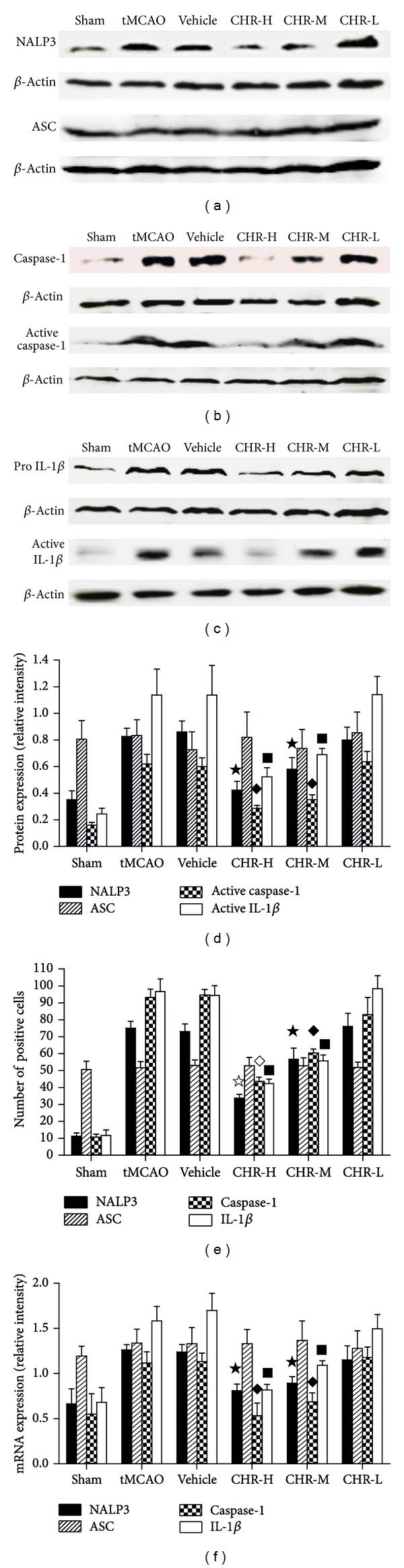
Effect of CHR on protein expression of NALP3, ASC (a), caspase-1, active caspase-1 (b), pro IL-1*β* and active IL-1*β* (c). For NALP3, active caspase-1 and active IL-1*β*, ^★^ *P* < 0.05, ^◆^ *P* < 0.05 and ^■^ *P* < 0.05 versus tMCAO and Vehicle (d). Bar graph of immunohistochemistry illustrating the expression of NALP3 (^*☆*^ *P* < 0.05 versus tMCAO, Vehicle and CHR-M, ^★^ *P* < 0.05 versus tMCAO, Vehicle), ASC, caspase-1 (^◊^ *P* < 0.05 versus tMCAO, Vehicle and CHR-M, ^◆^ *P* < 0.05 versus tMCAO, Vehicle) and IL-1*β* (^■^ *P* < 0.05 versus tMCAO and Vehicle) (e). Bar graph illustrating CHR reduced to mRNA of NALP3, caspase-1 and IL-1*β* (^★^ *P* < 0.05, ^◆^ *P* < 0.05 and ^■^ *P* < 0.05 versus tMCAO and Vehicle) (f).

To further investigate the impact of CHR on the endogenous expression of NALP3 inflammasome and IL-1*β* in ischemic hemisphere, the expression of NALP3, ASC, caspase-1, and IL-1*β* was also investigated in situ by immunohistochemical staining of tissue slices from the brain tissue slices from all experimental groups. The positively stained cells for the examined proteins were counted manually and intergroup comparisons were performed. The immunohistochemical staining of NALP3, ASC, caspase-1, and IL-1*β* of each group at 24 h after operation was shown in Figure 6. Few cells were expressing NALP3, caspase-1, or IL-1*β* in the cortex in Sham group indicating a low baseline of NALP3, caspase-1, and IL-1*β* expressions in the nonischemic cortex. In contrast, tissues from the tMCAO group showed an augmented expression of all three examined proteins. The number of cells expressing NALP3, caspase-1, or IL-1*β* in the ischemic cortex of tMCAO mice was significantly increased as compared with Sham group (*P* < 0.05). Similar results were also obtained from the Vehicle group and no significant difference was observed between the tMCAO and Vehicle groups (*P* > 0.05) (Figure 7(e)). In CHR-H and CHR-M groups, the number of positive cells for NALP3, caspase-1, or IL-1*β* was significantly lower than the tMCAO and Vehicle groups (*P* < 0.05) (Figure 7(e)). Then we further analyzed the protein expression of NALP3, ASC (Figure 7(a)), caspase-1 (Figure 7(b)), and IL-1*β* (Figure 7(c)) with Western blotting. It was found that the expressions of NALP3, caspase-1, and IL-1*β* at protein level were upregulated in tMCAO and Vehicle groups compared with Sham group (*P* < 0.05). High dose and middle dose CHR significantly decreased the NALP3, caspase-1, and IL-1*β* protein expression compared with tMCAO and Vehicle groups (*P* < 0.05) (Figure 7(d)). In agreement with the results of immunohistochemistry and Western blotting, the mRNA expression of NALP3, caspase-1, and IL-1*β* was upregulated in tMCAO and Vehicle groups compared with Sham group (*P* < 0.05). The overexpression of those factors was significantly decreased in CHR-H and CHR-M groups compared with tMCAO and Vehicle groups (*P* < 0.05) (Figure 7(f)).

## 4. Discussion

Although several treatments have been claimed to promote neuronal survival, reduce infarct volumes, and improve behavioral responses after experimental cerebral ischemia, the recombinant tissue plasminogen activator (rtPA) is the only FDA-approved drug for treatment of ischemic stroke clinically [28–30]. However, rtPA has a narrow window of efficacy of 3 h after the onset of stroke, which limits the use of this medicine only to a small portion of stroke patients. Since reperfusion after prolonged ischemia exaggerates tissue injury, in which inflammatory damage plays a critical role, anti-inflammatory oriented treatments may have the potential to mitigate the I/R-induced, inflammation-mediated secondary damage and improve the treatment outcome in stroke patients [31–35]. In this study, we have investigated the role of the NALP3 inflammasome in the inflammatory response induced by I/R using mouse tMCAO model, the most matured animal model that mimics human stroke [21]. Meanwhile, we examined the anti-inflammation and neuroprotective effects of CHR, a member of the anthraquinone family [36], in the mice who underwent tMCAO.

Previous pharmaceutical studies have shown that derivatives of anthraquinones exert multiple biological effects, including anticancer [37, 38], hepatoprotection [39], and antimicrobials [40]. A number of studies have also implied that extracts [41–43] containing CHR have a wide range of pharmacological activities, including anti-inflammation, antidyslipidemia, and antioxidant activities. It has been proved that CHR inhibits the nitric oxide production and activation of proinflammatory cytokines, such as tumor necrosis factor (TNF)-*α*, interleukin (IL)-6, NF-*κ*B, and caspase-1 in vivo and vitro [41]. Our results further confirmed the association between NALP3 inflammasome and the I/R induced brain damage. Furthermore, we firstly demonstrated that CHR could significantly suppress the inflammatory response after ischemic stroke, which is consistent with the previous studies reporting the effects of CHR on dextran sulfate sodium (DSS)-induced colitis and lipopolysaccharide (LPS)-induced inflammatory responses in mouse peritoneal macrophages [19].

NALP3 inflammasome is pivotal in the processing of active caspase-1 and downstream maturation of IL-1*β* [11]. IL-1*β* can then promote the increase of endothelial permeability and the expression of a number of adhesion molecules [32, 44]. These effects further result in the recruitment of inflammatory cells into the brain parenchyma, amplifying the inflammatory reaction within the ischemic brain. Our work focused on NALP3 since it is “apical” in the inflammation cascade and potentially an ideal target for anti-inflammation oriented therapies. A previous study has confirmed that NALP3 signaling is involved in liver I/R and silencing NALP3 can protect the liver from I/R injury [45]. The same results were observed during inflammation and immune responses in renal I/R as well [46]. Furthermore, NALP3 expression was elevated in the spinal cords during experimental autoimmune encephalomyelitis (EAE), and NALP3 (−/−) mice had a dramatically delayed course and reduced severity of disease [47].

In our study, we detected NALP3 inflammasome in both normal and ischemic brain tissues while the expression of NALP3 inflammasome was activated after cerebral I/R. We observed that NALP3, caspase-1, and IL-1*β* were all upregulated starting at an early stage after tMCAO and could remain at a high level for at least 72 h. For ASC, the adapter protein in the NALP3 inflammasome, its activation was relatively lagging behind and its expression would not be upregulated significantly until 72 h post-tMCAO. This somewhat unexpected discovery coincides with that of Abulafia et al. [48], Lindauer et al. [49], and Bostanci et al. [50], however,the causes need to be further researched. Furthermore, we demonstrated that systemic administration of CHR was effective in containing neurological impairment and tissue injury during cerebral I/R in a dose-dependent manner. We have shown that the neurological deficits, infarct volume, brain edema, and BBB disruption could all be significantly improved while the upregulation of NALP3, caspase-1, and IL-1*β* was significantly suppressed. All these are suggestive that the neuroprotective effect of CHR might involve inhibition of NALP3 inflammasome activation. Although it is still premature to conclude that inhibition of NALP3 inflammasome is the sole or foremost mechanism mediating the observed protective effects of CHR, our findings support the therapeutic effect of CHR in the acute phase of ischemic stroke.

## Figures and Tables

**Table 1:** Summary of the RT-PCR primers sequences.

Gene	Primers	Sequences
NALP3	Forward	5′-CGTGGTTTCCTCCTTTTGTATT-3′
	Reverse	5′-CGACCTCCTCTCCTCTCTTCTT-3′

ASC	Forward	5′-TCACAGAAGTGGACGGAGTG-3′
	Reverse	5′-TGTCTTGGCTGGTGGTCTCT-3′

Caspase-1	Forward	5′-CGTGGAGAGAAACAAGGAGTG-3′
	Reverse	5′-AATGAAAAGTGAGCCCCTGAC-3′

IL-1*β*	Forward	5′-TGAAATGCCACCTTTTGACAG-3′
	Reverse	5′-CCACAGCCACAATGAGTGATAC-3′
